# *Macropis fulvipes* Venom component Macropin Exerts its Antibacterial and Anti-Biofilm Properties by Damaging the Plasma Membranes of Drug Resistant Bacteria

**DOI:** 10.1038/s41598-017-16784-6

**Published:** 2017-11-29

**Authors:** Su Jin Ko, Min Kyung Kim, Jeong Kyu Bang, Chang Ho Seo, Tudor Luchian, Yoonkyung Park

**Affiliations:** 1 Department of Biomedical Science, Chosun University, Gwangju, 61452 Korea; 2 Division of Magnetic Resonance, Korea Basic Science Institute, Ochang, Chung-Buk, 363-883 Republic of Korea; 3 Department of Bioinformatics, Kongju National University, Kongju, 314-701 South Korea; 4 Department of Physics, Alexandru I. Cuza University, Iasi, Romania; 5 Research Center for Proteineous Materials, Chosun University, Gwangju, 61452 Korea

## Abstract

The abuse of antibiotics for disease treatment has led to the emergence of multidrug resistant bacteria. Antimicrobial peptides, found naturally in various organisms, have received increasing interest as alternatives to conventional antibiotics because of their broad spectrum antimicrobial activity and low cytotoxicity. In a previous report, Macropin, isolated from bee venom, exhibited antimicrobial activity against both gram-positive and negative bacteria. In the present study, Macropin was synthesized and its antibacterial and anti-biofilm activities were tested against bacterial strains, including gram-positive and negative bacteria, and drug resistant bacteria. Moreover, Macropin did not exhibit hemolytic activity and cytotoxicity to keratinocytes, whereas Melittin, as a positive control, showed very high toxicity. Circular dichroism assays showed that Macropin has an α-helical structure in membrane mimic environments. Macropin binds to peptidoglycan and lipopolysaccharide and kills the bacteria by disrupting their membranes. Moreover, the fractional inhibitory concentration index indicated that Macropin has additive and partially synergistic effects with conventional antibiotics against drug resistant bacteria. Thus, our study suggested that Macropin has potential for use of an antimicrobial agent for infectious bacteria, including drug resistant bacteria.

## Introduction

Since the discovery of penicillin, many antibiotics have been developed and used to treat infectious diseases caused by bacteria. Antibiotics kill bacteria by inhibiting cell wall synthesis, protein synthesis, or DNA synthesis. When an antibiotic is used in the clinic, consideration should be given to characteristics such as the antimicrobial range and mechanism of action. Nonetheless, the indiscriminate use of antibiotics has resulted in bacteria developing resistance to antibiotics. The number of resistant bacterial strains has increased, and some bacteria, referred to as superbugs, have emerged with resistance to most antibiotics, thus posing a serious health risk^1^. Therefore, there is a need to develop new drugs that are less likely to induce resistance to treat drug-resistant bacteria. Possible alternatives to conventional antibiotics are antimicrobial peptides (AMPs), which are part of the innate immune response^2^. AMPs have a broad spectrum of antimicrobial activity against bacteria, including multidrug resistant bacteria.

Generally, AMPs are found in nature and participate in host defense. AMPs are small proteins of between 12 and 50 amino acid residues. The secondary structures of AMPs include α-helices, β-sheets, extended structures, and loops^3^. In addition, AMPs tend to be amphipathic; i.e., they contain both hydrophilic and hydrophobic regions^4,5^. These characteristics promote the interaction of AMPs with the bacterial cell membrane, which comprises negatively charged lipids, such as phosphatidylglycerol (PG), phosphatidylethanolamine (PE), and cardiolipin (CL). Moreover, gram-negative bacteria contain lipopolysaccharide (LPS) in their outer membrane, and gram-positive bacteria have a peptidoglycan outside the bacterial plasma membrane, forming the cell wall. Different mechanisms of AMPs’ interaction with the membrane have been proposed and include the barrel stave model, the toroidal model, and the carpet model^3^. The barrel-stave model suggests that AMPs form pores by binding to the membrane surface. A hydrophilic region of the AMPs binds to the membrane’s lipid and hydrophobic regions, thus forming a channel^6^. The toroidal model is similar to the barrel-stave model; however, AMPs always contact the phospholipid head groups of the membrane. Peptides are then inserted into the membrane to form a pore^7^. In the carpet model, when a membrane reaches the threshold concentration of AMPs, it is disintegrated by the accumulation of peptides. AMPs then penetrate the membrane lipid layer to form a pore^8,9^. In addition, some AMPs inhibit DNA and protein synthesis^10^.

In many studies, treatment with two or more antibiotics revealed that this approach enhances the therapeutic effect, and it is now used extensively to treat diseases. For example, aminoglycoside and beta-lactam antibiotics have been tested in combination^11^ and with a β-lactamase inhibitor^12,13^. Combination therapy has a broader spectrum of bactericidal activity compared with that of a single antibiotic, reduces side effects, and decreases the risk of induced resistance. Recently, combinations of conventional antibiotics and AMPs were used to treat certain fungal and bacterial infections^14–16^.

In a previous study, a novel antimicrobial peptide named Macropin, with short α-helical structure, was isolated from venom of the solitary bee *Macropis fulvipes* (*Hymenoptera: Melittidae*) and was reported to have antimicrobial activity^17^. Bee venom, which comprises a complex mixture of active peptides, has been used for therapeutic purposes in various diseases and has immunological functions^18^.

In the present study, Macropin was tested for its antimicrobial activity against gram-negative and positive bacteria, including drug resistant bacteria. We also tested its cytotoxicity and hemolysis activities compared with those of melittin. Melittin is a well-known antimicrobial peptide from bee venom; however, it is highly cytotoxic^19^. The mechanism of action of Macropin was examined by n-phenyl-1-naphthylamine (NPN) uptake measurement and a 3,3′-dipropylthiadicarbocyanine iodide [DiSC_3_(5)] assay. Low vacuum scanning electron microscopy (SEM) was used to observe membrane destruction. Moreover, the synergistic effect of Macropin with conventional antibiotics was evaluated in a combination assay and by flow cytometry. Our results indicated that Macropin could potentially be used as an antimicrobial agent.

## Results

### Peptide synthesis

The Macropin sequence, theoretically calculated and observed molecular weights, retention time, hydrophobicity, hydrophobic moment, and net charge are summarized in Table 1. The observed molecular weight was consistent with the calculated molecular weight, indicating that the peptide was synthesized successfully. The wheel diagram represents the α-helical structure of Macropin (Fig. S1). The identity of the synthetic peptide was confirmed by reverse-phase high performance liquid chromatography (RP-HPLC) on a C18 column. The molecular weight of the peptide was verified by mass spectrometry (Fig. S2).

**Table 1:** Sequence and physicochemical properties of antimicrobial peptide Macropin.

Peptide	Sequence	Molecular weight		RT (min)	H	μM	charge
		Calculated	Observed				
Macropin	GFGMALKLLKKVL-NH_2_	1416.89	1416.9	28.943	0.645	0.564	+3

The value for hydrophobicity (H), hydrophobic moment (μM), and charge were obtained from http://heliquest.ipmc.cnrs.fr/. Mean retention time (RT, min) during reversed-phase high performance liquid chromatography (RP-HPLC).

### Minimum inhibitory concentration of macropin against microorganisms

The antimicrobial activities of AMP Macropin against gram-positive and negative bacteria are summarized in Table 2. Macropin showed antimicrobial activity against both gram-positive and negative bacteria in the concentration range of 3.13 μM to 25 μM. Macropin and nine antibiotics were then analyzed for their antimicrobial activity against drug-resistant bacteria. In the range of 2 to 32 μM, ciprofloxacin and levofloxacin showed activity against resistant *S*. *aureus* strains, whereas the other antibiotics showed no activity up to 128 μM. In the case of drug-resistant *P*. *aeruginosa* strains, tobramycin and gentamicin showed antimicrobial activity at concentrations of 32 μM and 64 μM (Table 3). Compared with the antibiotics, Macropin had stronger antimicrobial effects against drug-resistant bacterial strains.

**Table 2:** MIC of the antimicrobial peptide against microorganisms. ^a^Minimal inhibitory concentration (MIC) was determined as the lowest concentration of the peptide that inhibited growth.

Microorganisms	MIC(μM)^a^	
	Macropin	Melittin
**Gram positive**		
*Staphylococcus aureus* ATCC 25923	3.13	3.13
*Staphylococcus aureus* ATCC 29213	3.13	3.13
*Listeria monocytogenes* KCTC 3710	25	3.13
**Gram negative**		
*Pseudomonas aeruginosa* ATCC 27853	6.25	3.13
*Pseudomonas aeruginosa* ATCC 15692	6.25	3.13
*Escherichia coli* ATCC 25922	6.25	3.13

**Table 3:** MIC of peptide and antibiotics against *S*. *aureus* and *P*. *aeruginosa* strains.

Microorganism	Minimum inhibitory concentration (μg/mL)									
	Macropin	Ciprofloxacin	Levofloxacin	Gentamicin	Tobramycin	Oxacillin	Piperacillin	Ampicillin	Kanamycin	Cefotaxime
**Gram positive**										
*S*. *aureus* 95085	4.43	16	4	>128	>128	>128	>128	>128	>128	>128
*S*. *aureus* 691054	4.43	16	2	>128	>128	>128	>128	>128	>128	>128
*S*. *aureus* 254348	4.43	16	8	>128	>128	>128	>128	>128	>128	>128
*S*. *aureus* 547582	4.43	32	8	>128	>128	>128	>128	>128	>128	>128
*S*. *aureus* 771987	4.43	2	4	128	64	>128	>128	>128	>128	>128
*S*. *aureus* 949987	4.43	32	8	>128	>128	>128	>128	>128	>128	>128
**Gram negative**										
*P*. *aeruginosa* 3320	4.43	>128	>128	128	128	>128	>128	>128	>128	>128
*P*. *aeruginosa* 3318	4.43	>128	>128	64	64	>128	>128	>128	>128	>128
*P*. *aeruginosa* 1034	4.43	>128	>128	128	32	>128	>128	>128	>128	>128
*P*. *aeruginosa* 3241	2.21	128	>128	128	64	>128	>128	>128	>128	>128
*P*. *aeruginosa* 1162	4.43	>128	>128	64	32	>128	>128	>128	>128	>128
*P*. *aeruginosa* 3399	4.43	>128	>128	64	32	>128	>128	>128	>128	>128

### Cytotoxicity and hemolysis

**Figure 1:**
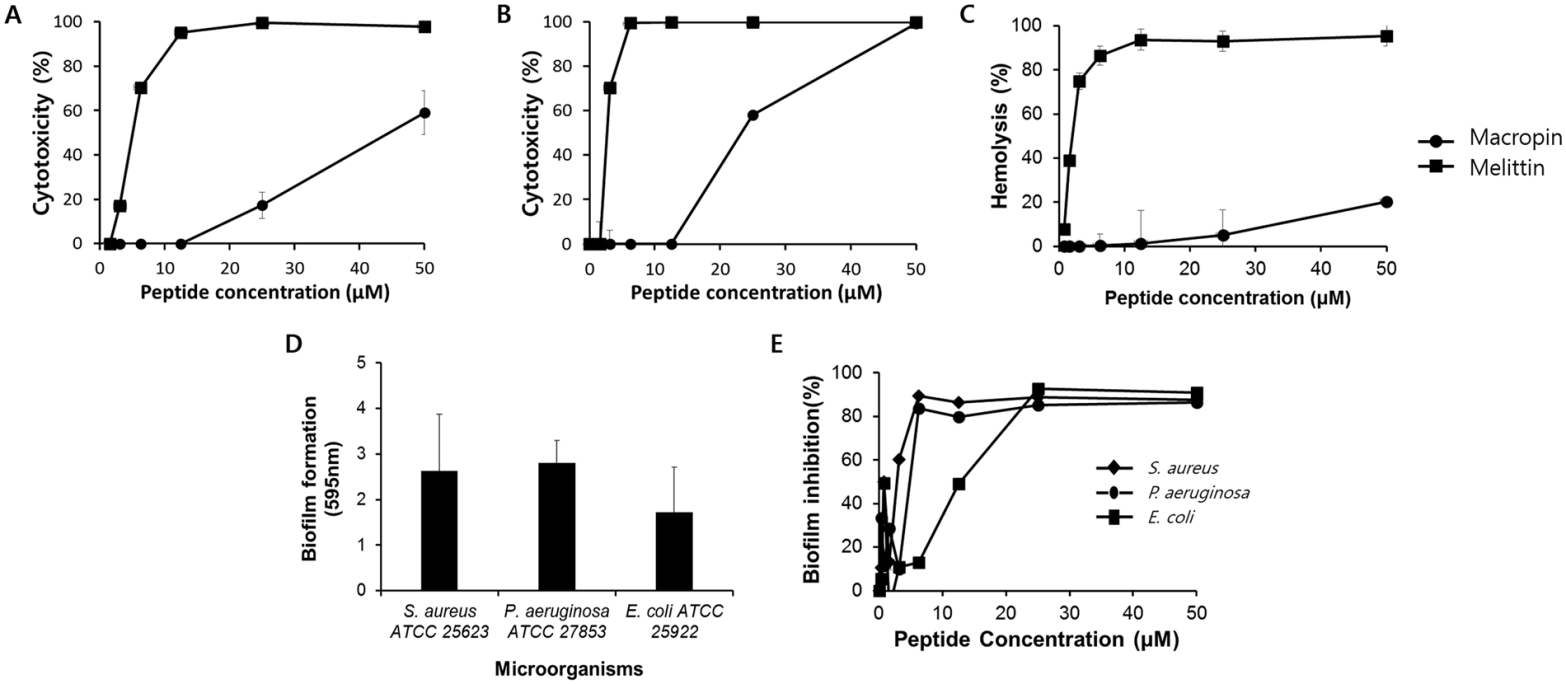
Cytotoxicity and Anti-biofilm Activity of Macropin. Cytotoxicity of peptide concentrations from 0 to 50 μM against (**A**) HaCaT cells, and (**B**) Raw 264.7 cells. After 24 h of incubation with the peptides, cytotoxicity was measured using an MTT (3-(4,5-dimethylthiazol-2-Yl)-2,5-diphenyltetrazolium bromide) assay. (**C**) Hemolytic activity of the peptide against red blood cells. The release of hemoglobin was measured using a microplate reader at 414 nm. (**D**) Biofilm formed by *S*. *aureus* ATCC 25923, *P*. *aeruginosa* ATCC 27853, and *E*. *coli* ATCC 25922. (**E**) Inhibition of biofilm formation by Macropin against the microorganisms. Each well contained 50 μL of the peptide and 50 μL of 5 × 10^5^ CFU/mL suspension of bacteria. The biofilms were then stained with crystal violet and absorbance was measured at 595 nm.

The cytotoxicity of Macropin was tested on HaCaT cells (keratinocytes) and Raw 264.7 cells (macrophages) using the MTT (3-(4,5-dimethylthiazol-2-Yl)-2,5-diphenyltetrazolium bromide) assay. At a concentration of 25 μM, Macropin produced cell survival rates of 82% and 41% in HaCaT and Raw cells, respectively (Fig. 1A and B). Melittin, a negative control, showed 100% toxicity at 12.5 μM in both cell lines. Hemolysis by Macropin was tested in an 8% suspension of red blood cells (RBCs) to test its toxicity to mammalian cells. The hemolysis rate of Macropin was approximately 5% at 25 μM. As negative control, Melittin was used because it is known to damage the membrane of RBCs. At 25 μM, melittin showed approximately 93% hemolytic activity (Fig. 1C). Thus, Macropin showed antimicrobial activity and low toxicity.

### Anti-biofilm activity

A biofilm is a social community of bacteria that produces multiple virulence factors. Bacteria in biofilms become more resistant to antibiotics, and biofilm-associated infections are difficult to treat. The degree of biofilm formation by the bacteria in the absence of the peptide was measured. Consequently, *S*. *aureus* ATCC 25923 and *P*. *aeruginosa* ATCC 27853 formed biofilms well (Fig. 1D). Next, we measured the inhibition of biofilm formation by Macropin. The maximal percentage of biofilm inhibition was 88%, 92%, and 84% against *S*. *aureus*, *P*. *aeruginosa*, and *E*. *coli*, respectively (Fig. 1E). We then evaluated the minimal biofilm inhibition concentration (MBIC) against drug-resistant strains of *S*. *aureus* and *P*. *aeruginosa*. In the concentration range of 12.5 μM to 50 μM, Macropin inhibited biofilm formation by *S*. *aureus* and *P*. *aeruginosa* (Table 4).

**Table 4:** Minimal biofilm inhibitory concentration (MBIC) of the Macropin against *S*. *aureus* and *P*. *aeruginosa* strains.

Microorganisms	MBIC(µM) Macropin
**Gram positive**	
*S*. *aureus* ATCC 25923	12.5
*S*. *aureus* 95085	25
*S*. *aureus* 691054	25
*S*. *aureus* 254348	50
*S*. *aureus* 254422	50
*S*. *aureus* 547582	50
*S*. *aureus* 771687	50
*S*. *aureus* 949987	50
**Gram negative**	
*P*. *aeruginosa* ATCC 27853	12.5
*P*. *aeruginosa* 1034	25
*P*. *aeruginosa* 3399	25
*P*. *aeruginosa* 3318	50
*P*. *aeruginosa* 3241	12.5
*P*. *aeruginosa* 3320	25
*P*. *aeruginosa* 1162	25

### Structure of the peptide and circular dichroism spectroscopy

**Figure 2:**
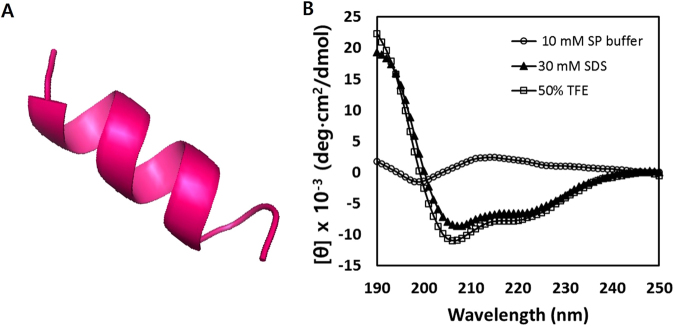
The structure analysis of the Macropin. (**A**) Three-dimensional structure simulations of Macropin. (**B**) Macropin formed an α-helical structure in 10 mM sodium phosphate buffer (pH 7.2), 30 mM SDS (mimicking microbial membrane environment), and 50% 2,2,2-trifluoroethanol (TFE) (mimicking the hydrophobic environment of the microbial membrane). The peptide concentration was fixed to 40 μM.

The secondary structure of Macropin in a membrane mimic environment was investigated using CD spectroscopy. In 10 mM sodium phosphate buffer (mimicking the aqueous environment), Macropin had a random coil structure. SDS and 2,2,2-trifluoroethanol (TFE) are widely used to mimic the cell membrane environment^20^. In 30 mM SDS (mimicking the microbial membrane environment) and 50% TFE (mimicking the hydrophobic environment of the microbial membrane), Macropin showed an α-helical conformation characterized by a positive band at 190 nm and negative bands at 208 and 222 nm (Fig. 2B). These results are consistent with the prediction that Macropin has an α-helical structure^17^, obtained using Mobyle@RPBS (Fig. 2A).

### Large unilamellar vesicle (LUV) aggregation

**Figure 3:**
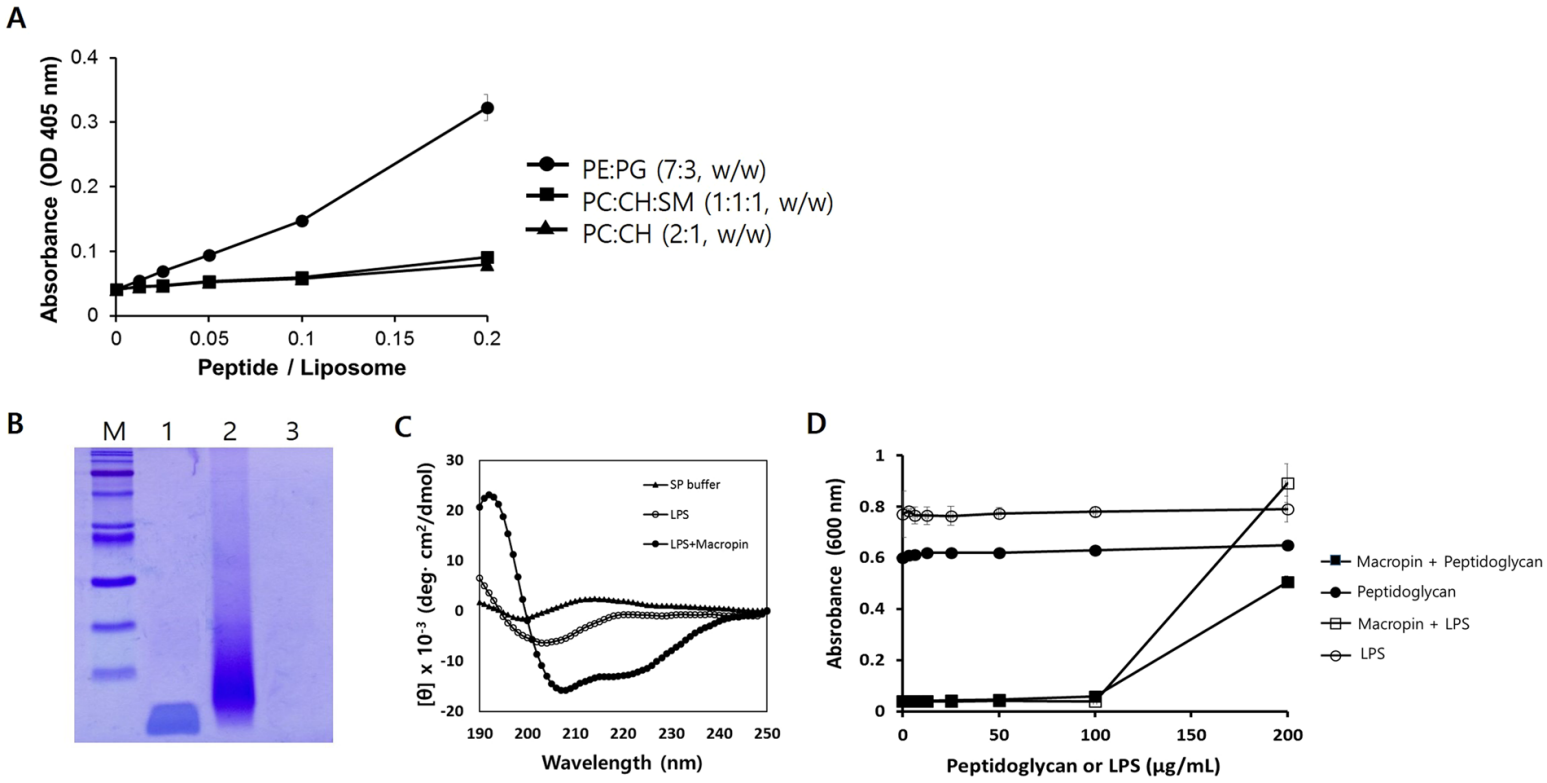
Aggregation of large unilamellar vesicles (LUVs) and Binding of the peptide to cell wall components. (**A**) A solution containing Macropin was added to 400 μM PC:CH (2:1, w/w), PE:PG (7:3, w/w), or PC:CH:SM (1:1:1, w/w). Liposome aggregation was monitored based on changes in the absorbance at 405 nm. PE, phosphatidylethanolamine; PG, phosphatidylglycerol; PC, phosphatidylcholine; CH; cholesterol; SM, sphingomyelin. (**B**) The peptide binds to the peptidoglycan of *S*. *aureus*. Lane 1: 5 μg of Macropin, lane 2: pellet of the mixture (5 μg of Macropin with 100 μg of peptidoglycan), lane 3: the supernatant of the mixture (5 μg of Macropin with 100 μg of peptidoglycan. (**C**) Binding affinity of the peptide for lipopolysaccharide (LPS), as measured using circular dichroism (CD) spectroscopy. Macropin (40 μM) was measured in the presence of 0.1% LPS. (**D**) Binding of Macropin to the peptidoglycan or LPS of the cell wall component was confirmed via antibacterial activity.

Liposome aggregation indicates a peptide-lipid interaction, and liposome turbidity served as a measure of the degree of aggregation caused by Macropin to PE:PG, phosphatidyl choline (PC):cholesterol (CH), and PC:CH: sphingomyelin (SM) systems. Macropin induced LUV aggregation in PE:PG, which is similar to a bacterial membrane. PE:PG turbidity was increased up to an absorbance of 0.3 at a peptide/lipid (P/L) ratio of 0.2 (Fig. 3A). By contrast, Macropin did not induce LUV aggregation of the PC:CH and PC:CH:SM systems.

### Binding of Macropin with cell wall components

To assess the ability of Macropin to bind to cell wall components, the peptidoglycan of *S*. *aureus* and the LPS of *P*. *aeruginosa* were used in binding assays. In sodium dodecyl sulfate polyacrylamide gel electrophoresis (SDS-PAGE) analysis, Macropin bound to the peptidoglycan was detected in the pellet of the mixture, but not in the supernatant (Fig. 3B). This result indicated that Macropin binds to the peptidoglycan, a cell wall component of gram-positive bacteria. In addition, we used CD spectra to study the conformational change caused by the interaction between the peptide and LPS. The CD spectra showed that Macropin has an α-helical conformation when interacting with LPS, a cell wall component of gram-negative bacteria (Fig. 3C). In previous studies, Macropin was found to bind to peptidoglycan and LPS. To confirm previous findings, Macropin’s antibacterial activity was measured by adding peptidoglycan into an *S*. *aureus* suspension and LPS into a *P*. *aeruginosa* suspension. As controls, only peptidoglycan or LPS was added to the culture medium of the bacteria. In the control, the bacteria grew constantly; however, in the experimental samples (peptide added), the absorbance of the mixture increased at 200 μg/mL of peptidoglycan or LPS (Fig. 3D). Blocking of the antibacterial activity of Macropin by peptidoglycan and LPS showed that peptide binds directly to a cell wall component.

### Membrane permeability

**Figure 4:**
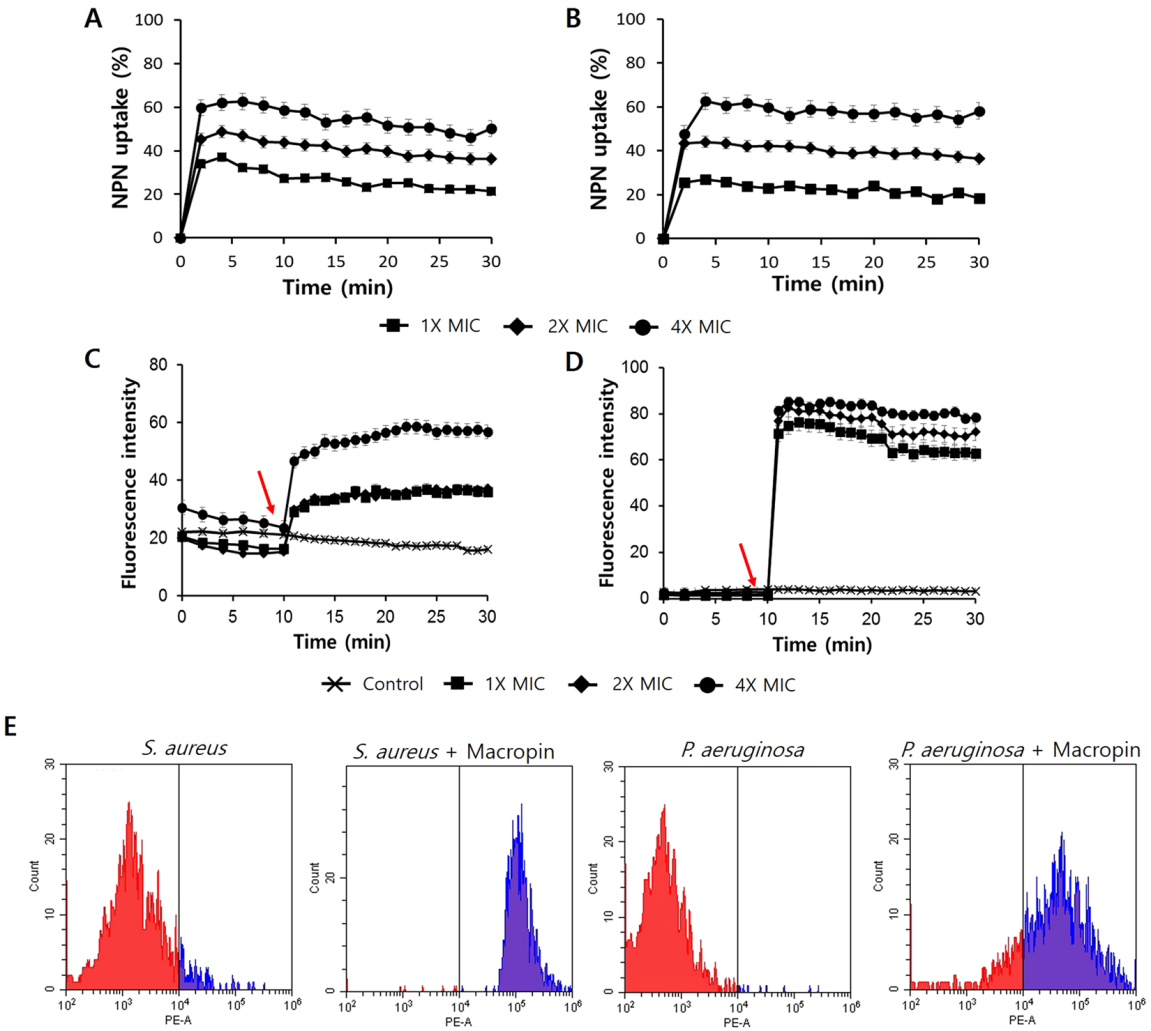
Effect of the peptide on the outer membrane and cytoplasmic membrane. Outer membrane permeabilization induced by Macropin as detected by n-phenyl-1-naphthylamine (NPN) uptake in (**A**) *S*. *aureus* ATCC 25923 and (**B**) *P*. *aeruginosa* ATCC 27853. The cytoplasmic membrane potential variation of (**C**) *S*. *aureus* and (**D**) *P*. *aeruginosa* treated by Macropin, and an assessment of the release of membrane potential sensitive dye DiSC_3_(5). The red arrows indicate the peptide-treated results, and control consisted of the buffer only (5 mM HEPES with 20 mM glucose and 100 mM KCl). (**E**) Flow cytometry analysis. *S*. *aureus* ATCC 25923 and *P*. *aeruginosa* ATCC 27853 were treated by 1 × the minimum inhibitory concentration (MIC) of Macropin for 1 h, and then stained with propidium iodide (PI). Macropin application resulted in increased fluorescence of 98.5% and 78.9% of *S*. *aureus* and *P*. *aeruginosa* cells, respectively.

An NPN uptake assay was performed to evaluate the ability of Macropin to permeabilize the outer membrane. The hydrophobic fluorescent probe NPN emits a weak fluorescent signal in an aqueous environment, but becomes strongly fluorescent in a hydrophobic environment when the cell membrane is disturbed^21^. Here, Macropin permeabilized the membrane in a dose-dependent manner in just a few minutes. After 5 min, NPN uptake increased to 60% in *S*. *aureus* and to 65% in *P*. *aeruginosa* at 4 × the MIC of Macropin. Thus, Macropin could permeabilize the outer membrane effectively (Fig. 4A and B).

The depolarization of the bacterial cytoplasmic membrane by Macropin was studied using the membrane potential-sensitive dye DiSC_3_(5). When the cytoplasmic membrane is depolarized and disrupted, DiSC_3_(5) is released into the medium and the fluorescence increases^22^. After stabilizing the bacteria with DiSC_3_(5) for 10 min, we added various concentrations of Macropin into each well of the plate. The fluorescence of DiSC_3_(5) increased compared with the control. As soon as we added the peptide, the fluorescence intensity increased by up to 70 arbitrary units (a.u.) in *S*. *aureus* and 100 a.u. in *P*. *aeruginosa*, whereas the fluorescence intensity of bacteria with DiSC_3_(5) without the peptide was 0 to 10 (Fig. 4C and D).

### Flow cytometry

Propidium iodide (PI) is an intercalating agent that binds to nucleic acids and is used evaluate to cell viability. PI stains nucleic acid after cell membrane disruption, resulting in an increase in fluorescent signals. Thus, cell membrane damage was measured by PI staining together with flow cytometry. In the absence of Macropin, 93.1% of *S*. *aureus* and 99% of *P*. *aeruginosa* cells showed no staining with PI, indicating intact cell membranes (viable cells). After peptide treatment, 98.5% and 78.9% of the cells showed PI fluorescence in *S*. *aureus* and *P*. *aeruginosa*, respectively (Fig. 4E).

### Low vacuum scanning electron microscopy (SEM)

**Figure 5:**
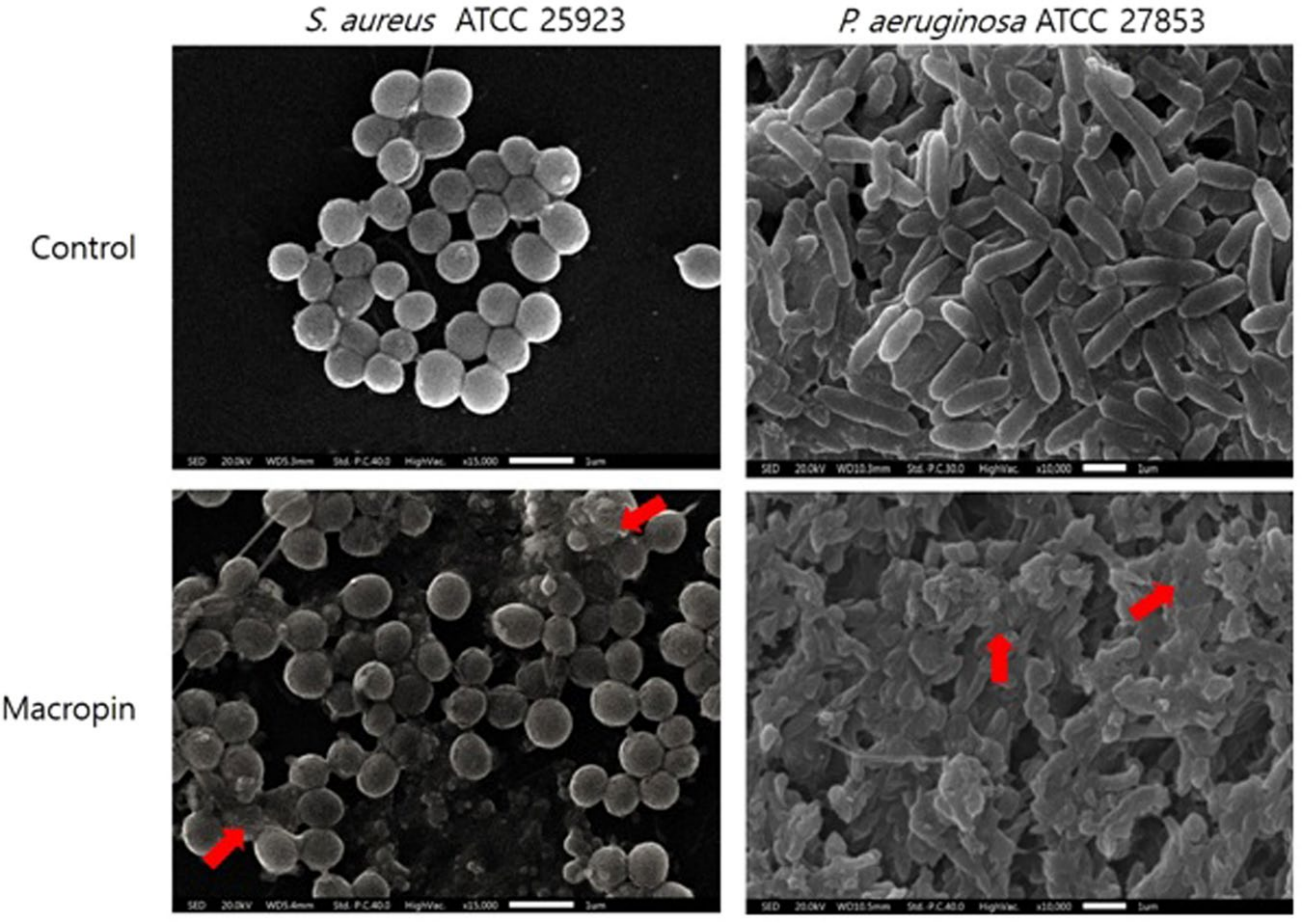
Low vacuum scanning electron microscopy (SEM) micrographs of peptide treated *S*. *aureus* and *P*. *aeruginosa*. The control bacteria without peptide were round and intact. *S*. *aureus* (ATCC 25923) and *P*. *aeruginosa* (ATCC 27853) were incubated with 1 × the minimum inhibitory concentration (MIC) of Macropin for 30 min. Macropin-induced changes in the bacterial membrane were observed.

This method was used to visualize the cell morphology after treatment of *S*. *aureus* and *P*. *aeruginosa* with Macropin. The control cells without Macropin showed a smooth surface. In contrast, treatment with 1 × the MIC of Macropin damaged the cell membrane. The membrane surface of *S*. *aureus* treated with Macropin showed blebs and damage. Part of the surface showed atrophy, destruction, and shrinking in *P*. *aeruginosa* treated with the peptide for 30 min (Fig. 5). These results suggested that Macropin induced the formation of membrane blebs on the surface of bacteria.

### Synergistic effect of Macropin and antibiotics

**Figure 6:**
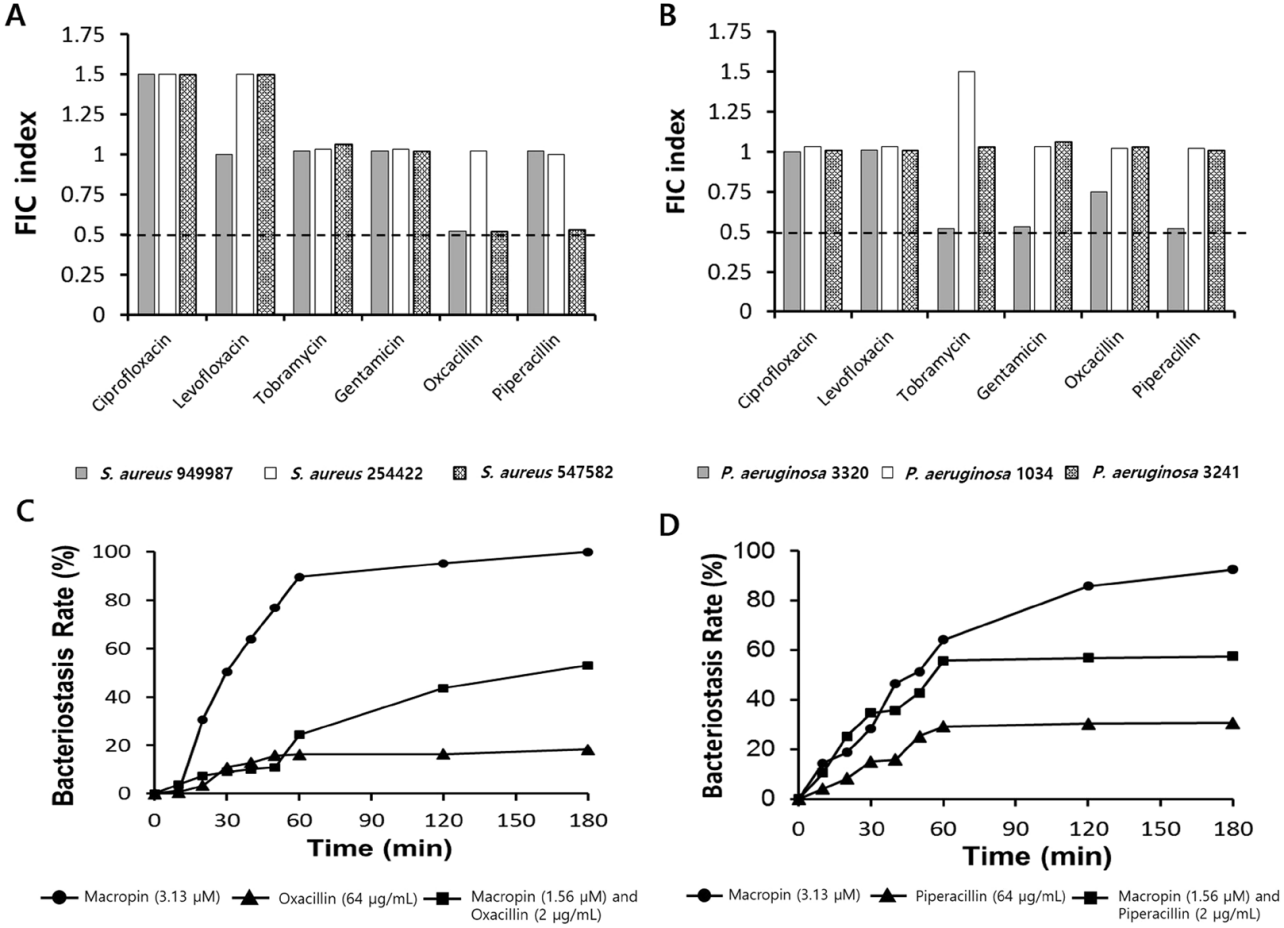
The activity of the peptide combined with traditional antibiotics against *S*. *aureus* and *P*. *aeruginosa* strains. Macropin combined with ciprofloxacin, levofloxacin, tobramycin, gentamicin, oxacillin, or piperacillin showed partial synergy or additive effects against (**A**) *S*. *aureus* strains and (**B**) *P*. *aeruginosa* strains. Especially, Macropin combined with oxacillin (in *S*. *aureus* 949987) and Macropin with piperacillin (in *P*. *aeruginosa* 3320) showed a partial synergy effect (FIC index 0.52). Fractional inhibitory concentration (FIC) indices were interpreted as follows: FICi ≤ 0.5 indicates synergy, 0.5 < FICi ≤ 1.0 indicates additive, 1 < FICi ≤ 4 indicates indifference, and FICi > 4 indicates antagonism. (**C**) The bacteriostasis rate represents the inhibition activity. Oxacillin and Macropin were used individually or in combination against *S*. *aureus* 949987. (**D**) Piperacillin and Macropin were used individually or in combination against *P*. *aeruginosa* 3320.

Combination therapy is used widely to treat diseases because of its better effect and lower costs. A combination of AMPs and antibiotics was found to have a better bactericidal effect than AMP or antibiotics alone. The *S*. *aureus* and *P*. *aeruginosa* strains used here are resistant to antibiotics; however, the addition of Macropin to the antibiotics improved the inhibition of bacterial growth. Combination therapy with Macropin and antibiotics showed antibacterial activity at a lower dose of the peptide or antibiotics compared with the peptide or antibiotics alone (Tables S1 and S2). The fractional inhibitory concentration (FIC) index indicated partial synergy and additive effects. When the *S*. *aureus* strains were treated with Macropin combined with gentamycin, tobramycin, ciprofloxacin, levofloxacin, piperacillin, or oxacillin, the FIC index revealed an additive effect. The combination of Macropin and oxacillin showed partial synergy (FIC index 0.52) against *S*. *aureus* 949987 (Fig. 6A). The *P*. *aeruginosa* strains treated with Macropin in combination with antibiotics showed the highest additive effect. In *P*. *aeruginosa* 3320, the combinations of Macropin with gentamycin, tobramycin, piperacillin, or oxacillin showed synergy (FIC index 0.52; Fig. 6B). Generally, the antibiotics and Macropin showed synergistic or additive effects.

### Bactericidal activity of the synergistic combinations

The combinations of Oxacillin and Macropin (for *S*. *aureus*) and Piperacillin and Macropin (for *P*. *aeruginosa*) increased the bacteriostasis rate rapidly up to 60 min, and approximately 90% of *S*. *aureus* 949987 and *P*. *aeruginosa* 3320 were killed at 180 min. The bacteriostasis rate increased gradually for bacteria treated with only the antibiotic. In the combination group treated with Macropin and antibiotics at less than the active concentration, the bacteriostasis rate increased more rapidly compared with rate observed on treatment with only antibiotics (Fig. 6C and D). Thus, Macropin accelerated the bacteriostasis rate; i.e., the number of living bacteria decreased.

### Inhibition of biofilm formation using the synergistic combinations

The anti-biofilm activity of Macropin, single antibiotics, and the combination groups were assessed using the MBIC. The MBIC values of Macropin against *S*. *aureus* and *P*. *aeruginosa* were 50 and 25 μM, respectively. All antibiotics used in experimental group showed no anti-biofilm activity at concentrations above 64 μg/mL. However, most combinations of the peptide and antibiotics improved the inhibition of biofilm formation. The MBIC value of the peptide and antibiotics decreased in combination groups. Macropin with oxacillin showed an additive effect (FIC index 0.63) against *S*. *aureus*. In the case of *P*. *aeruginosa*, the peptide with piperacillin, tobramycin, and gentamicin revealed strong synergistic effects, with FIC indices of 0.34, 0.26, and 0.63, respectively (Table 5).

**Table 5:** Minimal biofilm inhibition concentration (MBIC) and the FIC index of Macropin with antibiotics against *S*. *aureus* and *P*. *aeruginosa* strains.

MBIC in combination (μg/mL)				
Bacterial strains	Antibiotics		Macropin	FIC index
*S*. *aureus* 949987	Oxacillin	>64	70	0.63
*P*. *aeruginosa* 3320	Piperacillin	>64	35	0.34

Fractional inhibitory concentration (FIC) indices were interpreted as follows: FICi ≤ 0.5 indicates synergy, 0.5 < FICi ≤ 1.0 indicates additive, 1 < FICi ≤ 4 indicates indifference, and FICi > 4 indicates antagonism.

### Flow cytometry assessment of the synergistic groups

**Figure 7:**
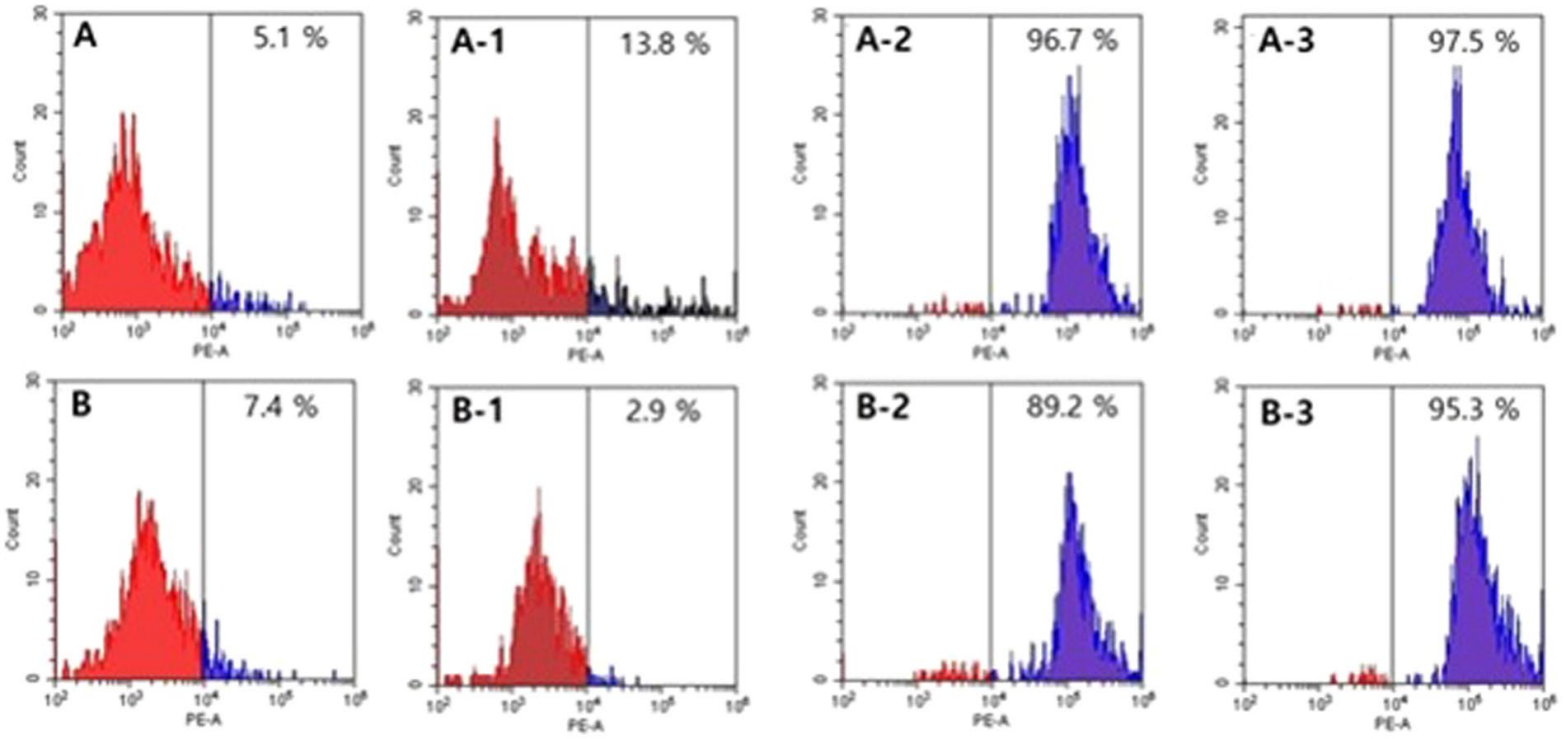
The combined effect as analyzed using flow cytometry analysis. (**A**) *S*. *aureus* 949987 and (**B**) *P*. *aeruginosa* 3320 were treated with the peptide, antibiotics, or both. (**A**) No peptide (5.1%). (A-1) oxacillin 64 μg/mL (13.8%), (A-2) Macropin 3.13 μM (96.7%), (A-3) Macropin 1.56 μM plus oxacillin 2 μg/mL (97.5%), (**B**) no peptide (7.4%), (B-1) piperacillin 64 μg/mL (2.9%), (B-2) Macropin 3.13 μM (89.2%), (B-3) Macropin 1.56 μM plus piperacillin 2 μM (95.3%).

The synergistic effects of the peptide and antibiotics were evaluated by flow cytometry. In the control, the bacterial cells were not stained, but in the experimental group, peptide treatment increased the PI fluorescence to 96.7% and 89.25% in *S*. *aureus* 949987 and *P*. *aeruginosa* 3320, respectively. When antibiotic treatment had no antimicrobial effect on the bacteria, the fluorescence of PI was only present in 13.8% of *S*. *aureus* and 2.9% of *P*. *aeruginosa* cells. In contrast, when the bacterial cells were treated with the combination of the peptide and the antibiotics, the percentage of PI staining increased. The combination of Macropin with oxacillin in *S*. *aureus* yielded PI staining in 97.5% of the cells. In case of *P*. *aeruginosa*, combinations of Macropin with piperacillin, tobramycin, or gentamicin were examined. The results revealed 95.3%, 88.7%, and 86% of PI, respectively. These data indicated that Macropin killed bacteria by disrupting the cell membrane, and the combination of Macropin with certain antibiotics had synergistic effects (Fig. 7).

### *In vivo* bactericidal effect of Macropin on *S*. *aureus*-infected mice

**Figure 8:**
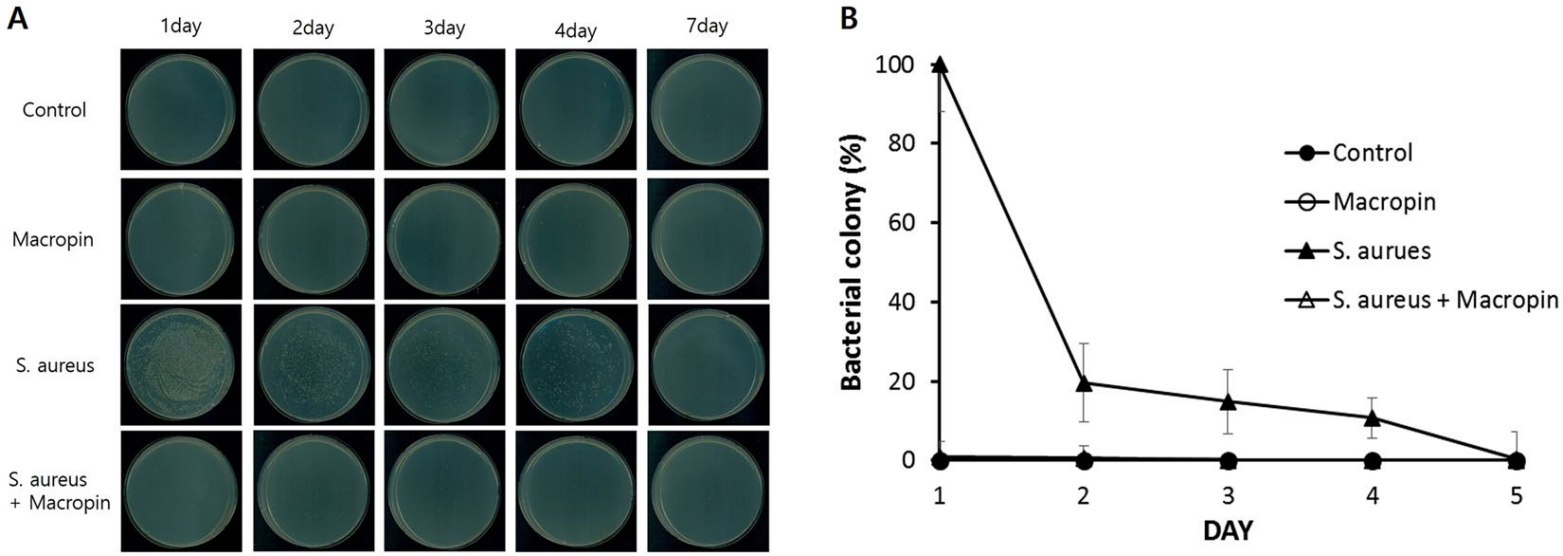
Effect of Macropin on *S*. *aureus* ATCC 25923 infected in the air pouch model *in vivo*. (**A**) Bacteria were cultured on agar plate overnight on 1, 2, 3, 4, 7 day after injection. (**B**) Bacterial colony is indicated as percentage in BALB/c mouse skin caused by *S*. *aureus* infection in the presence or absence of Macropin.

We further tested whether Macopin has therapeutic activity in an air pouch model. Anesthetized BALB/c mice were infected with *S*. *aureus* in the presence or absence of Macropin, and tissues were removed and cultured on an agar plate. The mice in the group treated with Macropin (1 mg/kg) showed a decrease in the number of cultured colonies compared to the mouse group treated with *S*. *aureus* only (Fig. 8). Thus, Macropin has antibacterial activity *in vivo*.

## Discussion

The prevalence of drug-resistant bacteria is increasing worldwide; therefore, there is a need to develop novel antimicrobial drugs. Additionally, diseases associated with biofilms, such as chronic infections and medical-appliance-related infections, are difficult to treat, which cause problems to public health that require novel solutions^23^. AMPs, a vital component of the innate immune system, represent novel therapeutic agents to treat microbial infections, because they are effective against pathogenic microorganisms and are less likely to induce drug resistance^24^. Although AMPs are attracting attention as a new drug, they have the disadvantage of high cost. Moreover, Melittin, a well-known peptide with strong antimicrobial activity from the bee venom, is cytotoxic at a concentration lower than the active concentration^25^. In a previous report, the AMP Macropin, from bee venom, was identified and reported to be composed of 13 amino acids^17^. Thus, Macropin is shorter than Melittin, which makes it more economical to synthesize. Therefore, in this study, we were interested in the possibility of Macropin being used drug.

Antimicrobial assays showed that Macropin possesses antimicrobial activity against both gram-positive and negative bacteria, including drug-resistant bacteria, especially *S*. *aureus* and *P*. *aeruginosa*. Furthermore, an assay of conventional antibiotics against resistant bacteria was conducted. Most of the traditional antibiotics used were ineffective against resistant bacteria. By contrast, Macropin showed antimicrobial activity against the resistant bacteria. Macropin’s hemolytic activity on RBCs and cytotoxicity toward keratinocytic (HaCaT) and macrophagic cells (Raw 264.7) were lower than those of melittin, which is a representative peptide derived from bees^26^. The active concentration of Macropin is from 3.13 μM to 6.25 μM, similar to that of Melittin, and the cytotoxicity effect of Macropin was reported at a concentration of 25 μM, which is about 8-times the active concentration. On the other hand, Melittin showed hemolysis and cytotoxicity at a concentration lower than the active concentration of 3.13 μM. Macropin displayed antimicrobial activity at a concentration similar to the active concentration of Melittin and was not cytotoxic up to about 10-times the active concentration. In addition, Macropin could inhibit biofilm formation in *S*. *aureus* and *P*. *aeruginosa* strains, including resistant bacteria, at concentrations ranging from 12.5 μM to 50 μM. Thus, we demonstrated that Macropin is a suitable antimicrobial agent and must be tested against relevant infections in humans.

Most AMPs have one of four types of secondary structures: an α-helix, a β-sheet, an extended structure or a loop type structure. CD spectra are used to determine the secondary structure of a peptide. CD assays performed in the present study showed that Macropin appeared as a random coil in aqueous solution, whereas in membrane-mimicking environments, such as SDS and TFE solutions, it formed an α-helix. LUVs are often used as a model of the bacterial membrane to study the interaction between a cationic peptide and the membrane. Prokaryotic and eukaryotic cells differ in the composition of their cellular membranes. For example, PC and PE have no net charge, whereas SM and cholesterol are neutral (not charged). PG and PS have a net negative charge^27^. The cell membrane of bacterial pathogens comprises PG and PS. In contrast, the membrane of human cells, such as erythrocytes, is enriched in PC, PE, and SM. Macropin induced aggregation of the PE:PG liposome (in the bacterial membrane) but not of the PC:CH (erythrocyte) and PC:CH:SM (mammalian cell) liposomes. These results correlated with the hemolytic and cytotoxicity properties of Macropin, suggesting that Macropin has cell selectivity for negative bacterial membranes compared to mammalian cell membranes.

AMPs are attracted to bacterial membranes via electrostatic bonding between the cationic peptide and the negatively charged membrane surface. LPS is the major component on the outer leaflet of the outer membrane of gram-negative bacteria^28^. The binding of LPS by AMPs, which plays an important role in killing bacteria, might proceed via an amphipathic sequence. AMPs that can bind to LPS and disrupt the membrane have attracted increased interest in terms of drug development^29^. Gram-positive bacteria possess structural components such as a thick peptidoglycan layer and lipoteichoic acid. These components perform permeability barrier functions, and the ability of a peptide to bind to such a cell wall component is a prerequisite for antibacterial activity. After a peptide binds to a cell wall component through electrostatic interaction, an AMP could be inserted into the bacterial membrane^30^. While binding to the cell wall, the peptide comes into contact with the cytoplasmic membrane. Macropin binds to the peptidoglycan of *S*. *aureus* and to the LPS of *P*. *aeruginosa*. To examine the mechanism of action of Macropin, outer membrane permeability and cytoplasmic membrane depolarization assays were performed. Macropin induced an increase in NPN and DiSC_3_(5)-related fluorescence. Our experiments confirmed that Macropin disturbs the outer membrane and depolarizes the cytoplasmic membrane. Furthermore, flow cytometry analysis indicated that Macropin can destroy a bacterial membrane, allowing PI to bind nucleic acids. Observation of morphological changes using low-vacuum SEM confirmed that the Macropin caused damage to the bacterial membrane. This agreed with a previous study that showed that the bacterial membrane is disrupted by AMPs such as LL37^31^ and melittin^25^, and that this action causes a loss of functional integrity of the membrane^32^. In particular, melittin is a peptide derived from bees and exerts a bactericidal effect by damaging the bacterial membrane. In this paper, the superiority of Macropin compared with melittin showed that the activity of Macropin is similar but its cytotoxicity is lower.

Traditional antibiotics exert their antimicrobial activity by inhibiting cell wall synthesis and the transcription and replication of DNA. However, mutations in the components of these mechanisms result in drug-resistant bacteria^33,34^. Compared with antibiotics, AMPs have several unique characteristics, such as a wide range of antibacterial activities against antibiotic-resistant pathogens and a relatively low rate of mutation induction in bacteria^35^. Thus, AMPs are promising antimicrobial agents that can be used to treat several infectious diseases, either alone or in combination with traditional antibiotics. Using killing tests and antimicrobial activity assessments of the combination group, we observed synergistic effects of the combination of Macropin with traditional antibiotics. The results of this study showed that the Macropin-Oxacillin and Macropin-Piperacillin combinations have an additive synergy for *S*. *aureus* 94998 and *P*. *aeruginosa* 3320, respectively. Furthermore, Macropin and antibiotic combinations inhibited biofilm formation of drug-resistant bacteria. The Macropin-Oxacillin and Macropin-Piperacillin combinations displayed synergistic activity against biofilm formation by *S*. *aureus* and *P*. *aeruginosa*, respectively. The decrease in MBIC concentration of the peptide combined with antibiotics indicated that it can inhibit biofilm formation successfully^36^. The synergistic action of the peptide and antibiotic offers an alternative strategy to treat biofilm-related infections. The mechanisms of the synergistic effect of the combination are still unclear. One of the proposed mechanisms is that the AMP increases the permeability of the cell membrane and facilitates the entry of antibiotics into the cytoplasm to access the intracellular target^37^. Thus, a combination of the peptide and traditional antibiotics can improve their antimicrobial activity, which suggested that combination therapy could be used against resistant bacteria.

In conclusion, Macropin binds to negatively charged components, such as peptidoglycan or LPS, of bacterial membranes and then kills the bacteria through membrane disruption and cell penetration. Macropin forms an α-helix structure in a bacteria-mimicking environment. In addition, Macropin can inhibit the growth of drug-resistant bacteria and biofilm formation, but has little or no hemolytic activity or cytotoxicity toward mammalian cells at microbiologically effective concentrations. To verify the effect of Macropin *in vivo*, we performed an air pouch model experiment in mice, which demonstrated that Macropin has antibacterial activity *in vivo*. Furthermore, combinations of the peptide and antibiotics have synergistic effects against certain drug-resistant bacteria. These effects can increase antimicrobial activity and help delay the emergence of drug resistance. Consequently, Macropin is a promising candidate for an antimicrobial drug to treat bacterial infection and as part of a combination therapy with antibiotics against multi-antibiotic-resistant bacteria.

## Materials and Methods

### Materials

Rink amide 4-mehylbenzhydrylamine resin, fluoren-9-ylmethoxycarbonyl (Fmoc), amino acids, and other reagents for peptide synthesis were purchased from Calbiochem-Novabiochem (La Jolla, CA, USA). L-α-phosphatidylethanolamine (PE), sphingomyelin (SM), cholesterol (CH), L-α-phosphatidylglycerol (PG), and egg yolk L-α-phosphatidylocholine (PC) were obtained from Avanti Polar Lipids (Alabaster, AL, USA). LPS (from *P*. *aeruginosa*), peptidoglycan (from *S*. *aureus*), 3,3′-Dipropylthiadicarbocyanine iodide [DISC_3_(5)], *n*-phenyl-1-naphtylamine (NPN), propidium iodide (PI), ciprofloxacin, levofloxacin, gentamicin, kanamycin, tobramycin, cefotaxime, oxacillin, erythromycin, ampicillin, piperacillin, and vancomycin were obtained from Sigma-Aldrich (St Louis, MO, USA). *Staphylococcus aureus* ATCC 25923, *Staphylococcus aureus* ATCC 29213, *Escherichia coli* ATCC 25922, *Pseudomonas aeruginosa* ATCC 27853, and *Pseudomonas aeruginosa* ATCC 15692 were obtained from the American Type Culture Collection (ATCC). *Listeria monocytogenes* KCTC 3710 was obtained from the Korea Collection for Type Cultures (KCTC). *Staphylococcus aureus* 95085, 691054, 254348, 254422, 547582, 771687, and 949987 were antibiotic resistant strains isolated from hospital patients with cholelithiasis. *Pseudomonas aeruginosa* 3320, 3318, 1034, 3241, 1162, and 3399 were antibiotic resistant strains isolated from patients with otitis media in Chonnam National University hospital. The patients provided informed consent for the use of their clinical isolate.

### Peptide synthesis

The peptide was synthesized according to a solid-phase-9-fluorenylmethoxycarbonyl1 (Fmoc) method on a Rink amide 4-methylbenzhydrylamine resin using a Liberty microwave peptide synthesizer (CEM Co. Matthews). Then, 0.1 M N-hydroxy benzotriazole in piperidine and dimethylformamide (DMF), 0.45 M 2-(1H-benzotriazole-1-yil)-1,1,3,3-tetramethyluroniumhexafluorophosphate in DMF and 2 M N,N-diisopropyl ethylamine in N-methylprrolidone were used as linkage reagents. After washing with dichloromethane (DMF), cleavage was performed in a solution of trifluoroacetic acid, phenol, water, and trisopropylsaline for 2 h at room temperature. Finally, the dried peptide was purified by reverse-phase high-performance liquid chromatography (RP-HPLC) on a Jupiter C18 column (4.6 × 250 mm, 300 Å, 5 μm). The molecular mass of the peptide was confirmed using a matrix-assisted laser desorption/ionization time-of-flight mass spectrometer (Kratos Analytical Inc., Chestnut Ridge, NY, USA).

### Antibacterial activity assay

The antibacterial activity of the peptide was determined using its minimal inhibitory concentration (MIC). Gram positive and gram negative bacteria, and drug resistant bacteria strains were cultured overnight at 37 °C in Mueller-Hinton broth (MHB)^38^. The bacteria were diluted to a final concentration 2 × 10^5^ colony-forming units (CFU)/mL in fresh MHB. The peptides and commercial antibiotics were diluted in 10 mM sodium phosphate buffer (pH 7.2). The bacteria was mixed with serially diluted peptide, or antibiotics in a 96 well plate and incubated at 37 °C for 18 h. After that, growth was measured by absorbance at 600 nm. The MICs were determined as the lowest concentration that inhibited bacterial growth. The result is represented as an average of the MIC obtained from three independent experiments.

### Cell culture and cytotoxicity

HaCaT and Raw 264.7 cells were seeded in a 96-well plate at 10^4^ cells/well and incubation for 24 h. The peptide was added to wells, and then incubated for 24 h. Then, 0.5 mg/mL MTT was added to each well and incubated 4 h at 37 °C. The supernatants were aspirated, and DMSO was added to dissolve the formazan crystals. Cytotoxicity was measured using microplate reader at 570 nm. The test was reproduced at least three times using two replicates.

### Hemolytic activity

Red blood cells (RBCs) at a final concentration of 8% were mixed with the peptide in a 96-well plate and incubated for 1 h at 37 °C. Thereafter, the plate was centrifuged at 1500 × *g* for 10 min. The supernatant was transferred to a new 96-well plate and its absorbance was monitored at 414 nm. In the controls, 100% hemolysis consisted of RBC in 0.1% Triton X-100, whereas zero hemolysis control consisted of RBCs in PBS (pH 7.4). Each measurement was performed at least three times using two replicates. Percent hemolysis was calculated according to the following equation^39^:

$$\begin{array}{rcl} \% {\rm{Hemolysis}} & = & [({{\rm{Abs}}}_{414}\,{\rm{in}}\,{\rm{the}}\,{\rm{peptide}}\,{\rm{solution}}-{{\rm{Abs}}}_{414}\,{\rm{in}}\,{\rm{PBS}})\\  &  & \div\,({{\rm{Abs}}}_{414}\,{\rm{in}}\,0.1 \% \,{\rm{Triton}}\,{\rm{X}} \mbox{-} 100-{{\rm{Abs}}}_{414}\,{\rm{in}}\,{\rm{PBS}})]\times 100\end{array}$$

### Biofilm inhibition assay

Bacteria were diluted to a final concentration 5 × 10^5^ CFU/mL in fresh MHB containing 0.2% of glucose. The bacteria was mixed with peptide in a 96-well plate and incubated. Thereafter, the biofilm was fixed with 100% methanol for 15 min, then aspirated and the wells were dried. Wells were stained with 0.1% crystal violet for 2 h. The crystal violet was removed and the wells were rinsed with distilled water. After air-drying, 95% ethanol was added to dissolve the biofilm, and the absorbance was measured using a microplate reader at 595 nm. The minimal biofilm inhibitory concentration (MBIC) was determined as the lowest concentration that inhibited the biofilm formation. Each measurement was performed at least three times using two replicates.

### Circular dichroism (CD) spectroscopy

The CD spectra were recorded for 40 μM peptide in a buffer consisting of 10 mM sodium phosphate, 30 mM sodium dodecyl sulfate (SDS), and 50% 2,2,2-trifluoroethanol (TFE). The CD spectra were acquired on a Jasco 810 spectropolarimeter (Jasco, Tokyo, Japan) equipped with a quartz cell with a 0.1-cm path length. The spectra were recorded at wavelengths from 190 nm to 250 nm.

### Preparation of liposome and liposome aggregation

Large unilamellar vesicles (LUV) were prepared by the freeze-thaw method^40^. The lipid concentration was determined using a standard phosphate assay^41^. Liposome aggregation was monitored by measurement of absorbance. The peptide at 5, 10, 20, 40, and 80 μM was added to 400 μM LUVs of PC:CH (10:1, w/w), PE:PG (7:3. w/w), or PC:CH:SM (1:1:1, w/w). The increase absorbance was measured at 405 nm, using a microplate reader.

### Peptide binding to cell wall components

The insoluble peptidoglycan was added to 10 mM Tris-HCl (pH 7.5), containing 5 μg of peptide, and was incubated for 1 h. The supernatant and pellet were then subjected separately to SDS-PAGE in a 15% gel. The gel was stained for 30 min with a staining solution and then destained with destaining solution. The peptide in the presence or absence of LPS (0.1%) was used in a binding assay examined by CD spectroscopy. Peptidoglycan or LPS, peptide, and peptide and bacteria were incubated overnight. Then, absorbance is measured at 600 nm.

### Outer membrane permeabilization assay

Outer membrane permeability was determined based on the uptake of NPN, a fluorescent dye. The bacteria were re-suspended to an optical density at 600 nm of 0.2 in 5 mM HEPES (pH 7.2). The bacteria and peptide were mixed with NPN solution (final concentration 10 μM) in a black 96-well plate. 0.1% Triton X-100 was used as a negative control. Each measurement was performed at least three times using two replicates.

### Cytoplasmic membrane depolarization assay

Membrane depolarization by the peptide was quantified using a membrane-sensitive fluorescent dye, 3,3′-Dipropylthiadicarbocyanine Iodide [DiSC_3_(5)]. The bacteria were cultured and washed with washing buffer (5 mM HEPES and 20 mM glucose) three times, and re-suspended to an OD_600_ of 0.05 in buffer (5 mM HEPES, 20 mM glucose, and 100 mM KCl). The bacteria was incubated with at final concentration 1 μM DiSC_3_(5) solution for 1 h. Then, a solution of the peptides was added to the mixture of bacteria and DiSC_3_(5). Each measurement was performed at least three times using two replicates.

### Flow cytometry

Antibiotics, and a mixture of peptide and antibiotics was incubated with the bacteria (OD_600_ of 0.5) for 1 h. The cells were harvested by centrifugation, washed, and incubated with propidium iodide (PI) at a final concentration 10 μg/mL for 30 min at 4 °C. Thereafter, the unbound dye was removed by washing with PBS.

### Low vacuum scanning electron microscopy (SEM)

The bacteria cells (OD_600_ of 0.2) were incubated with peptide for 30 min at 37 °C. After that, the cells were fixed 2.5% glutaraldehyde in PBS at 4 °C overnight. The fixed samples were washed with PBS and post-fixed with 1% osmium tetroxide (OsO4) for 1 h. Then, the cells were dehydrated in 50, 60, 70, 80, 90, and 100% ethanol for 15 min each. Each sample was placed on a cover glass and coated with platinum and then examined under a low vacuum scanning electron microscope (JEOL JSM-IT300, Japan).

### Synergy activity

The synergistic effects of Macropin and traditional antibiotics were assessed according to the broth microdilution checkerboard method^42^. Macropin, gentamycin, tobramycin, ciprofloxacin, levofloxacin, oxacillin, and piperacillin were diluted in 10 mM sodium phosphate buffer (pH 7.2). Then, each of different concentration of antibiotics and peptide was mixed and added into bacteria in a 96-well plate. The result represents an average of the FIC index obtained from three independent experiments. The effects of this combination were assessed using the fractional inhibitory concentration index (FIC index):

$$\begin{array}{rcl}{\rm{FIC}}\,{\rm{index}} & = & {\rm{FICA}}+{\rm{FICB}}\\  & = & ({\rm{MIC}}\,{\rm{of}}\,{\rm{drug}}\,{\rm{A}}\,{\rm{in}}\,{\rm{combination}}/{\rm{MIC}}\,{\rm{of}}\,{\rm{drug}}\,{\rm{A}}\,{\rm{alone}})\\  &  & +\,({\rm{MIC}}\,{\rm{of}}\,{\rm{drug}}\,{\rm{B}}\,{\rm{in}}\,{\rm{combination}}/{\rm{MIC}}\,{\rm{of}}\,{\rm{drug}}\,{\rm{B}}\,{\rm{alone}})\end{array}$$

The FIC index was represented as follows: FICi ≤ 0.5 indicated synergy, 0.5 < FICi ≤ 1 indicated additive, 1 < FICi ≤ 4 indicated indifference, FICi > 4 indicated antagonism^43^.

### Time-dependent killing of the synergistic group

The peptide and antibiotics were mixed with bacteria. Every 10 min, the mixture was plated onto MHB agar. The bactericidal activity of the antimicrobial agent was evaluated by the bacteriostasis rate as follows^44^;

$$\begin{array}{rcl}{\rm{Bacteriostasis}}\,{\rm{rate}} & = & ({\rm{colony}}\,{\rm{number}}\,{\rm{of}}\,{\rm{the}}\,{\rm{negative}}\,{\rm{control}}\\  &  & -\,{\rm{colony}}\,{\rm{number}}\,{\rm{of}}\,{\rm{test}}\,{\rm{group}})\\  &  & \div\,{\rm{colony}}\,{\rm{number}}\,{\rm{of}}\,{\rm{negative}}\,{\rm{control}}\times 100 \% \end{array}$$

### Effects of Macropin *In vivo*

Six- to seven-week-old female BALB/c mice (20 ± 0.5 g) were used in this study. The back skin of the mice was using a razor blade. The mice were anesthetized with isoflurane and air cavities were produced by infecting 3 mL of air in the back^45^. The mice were then infected with 1  × 10^8^ CFU/mL of *S*. *aureus* ATCC 25923 in the presence or absence of 1 mg/kg Macropin. Control was injected with PBS. On days 1, 2, 3, 4, and 7, tissues surrounding the air pouch were harvested and homogenized separately. The homogenates were plated on MHB agar plates. The plates were incubated at 37 °C overnight and then the colonies were counted.

### Ethics Statement

This study conformed to the ethical standards of the Institutional Ethics Committee of Chosun University, and the protocol was approved by the Institutional Ethics Committee of Chosun University. All experiments were carried out in strict accordance with the National Institutes of Health Guidelines for the Ethical Treatment of Animals and the guidelines of the Center for Experimental Animals of Chosun University for Medical Science (Permit number: CIAUCUC2017-S0025).

## Electronic supplementary material

Supplementary data
