# Supplementary material for: Macropis fulvipes Venom component Macropin Exerts its Antibacterial and Anti-Biofilm Properties by Damaging the Plasma Membranes of Drug Resistant Bacteria
Source: Sci Rep. 2017 Nov 29;7:16580. doi: 10.1038/s41598-017-16784-6 (PMC5707368; doi:10.1038/s41598-017-16784-6)
Supplement: Supplementary file 1 — Supplementary data [file 41598_2017_16784_MOESM1_ESM.pdf]

*Supplementary data*

# *Macropis fulvipes* Venom component Macropin Exerts its Antibacterial and Anti-Biofilm Properties by Damaging the Plasma Membranes of Drug Resistant Bacteria

**Su Jin Ko[1], Min Kyung Kim[1], Jeong Kyu Bang[2], Chang Ho Seo[3], Tudor Luchian[4,*], Yoonkyung Park[1,5*]**

[1]Department of Biomedical Science, Chosun University, Gwangju 61452, Korea. [2]Division of Magnetic Resonance, Korea Basic Science Institute, Ochang, Chung-Buk 363-883, Republic of Korea. [3]Department of Bioinformatics, Kongju National University, Kongju 314-701, South Korea. [4]Department of Physics, Alexandru I. Cuza University, Iasi, Romania. [5]Research Center for Proteineous Materials, Chosun University, Gwangju 61452, Korea.

[*]Corresponding authors (y_k_park@chosun.ac.kr and luchian@uaic.ro)

**Conflict of interest:** The authors declare that no conflict of interest exists.

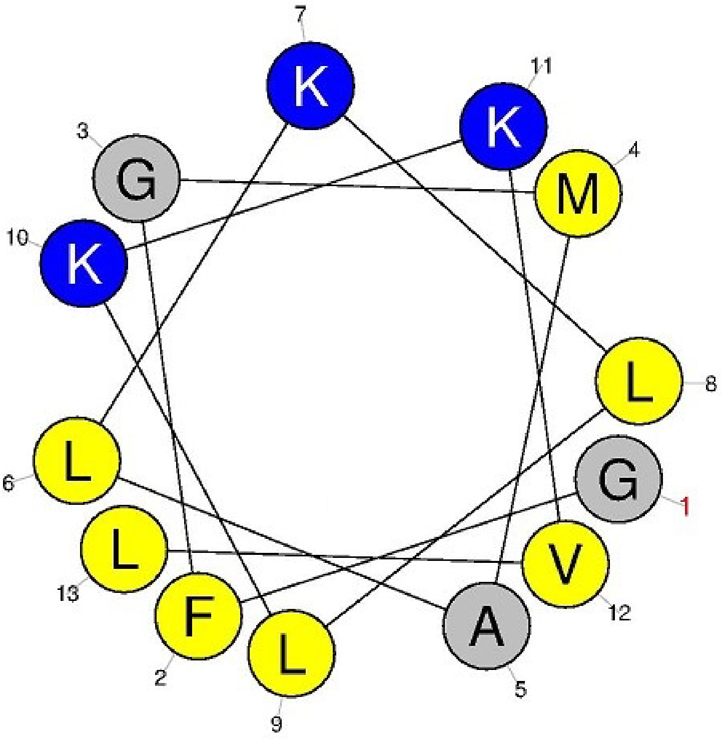


**Figure S1. Helical wheel diagram for Macropin**. The projections was obtained from http://heliquest.ipmc.cnrs.fr/cgi-bn/ComputParam.py. Macropin consists of 13 amino acids, starting with glycine (Gly, G) and ending with leucine (Leu, L). G: Glycine, F: Phenylalanine, M: Methionine, A: Alanine, L: Leucine, K: Lysine, V: Valine.

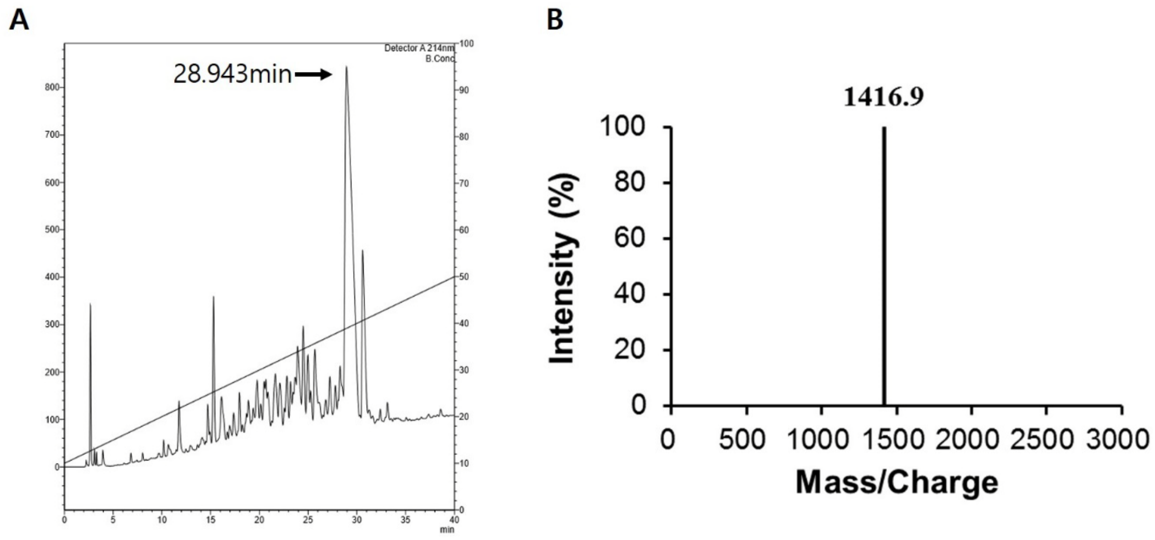


**Figure S2**. **RP-HPLC and mass spectrometry.** (A) Reversed-phase high performance liquid chromatography (RP-HPLC) profile on a C18 column with detection at 214 nm. The black arrow represents the retention time of Macropin (28.943 min). (B) Matrix-assisted laser desorption/ionization (MALDI) mass spectrometric analysis of the synthetic peptide. The major peak appeared at the m/z value of Macropin (1416.9).

**Table S1. Effect of Macropin in combination with antibiotics against *S. aureus* strains.**

| Peptide-Antibiotic | Best Concentration of Mixture (MIC, µg/mL) | | Peptide (MIC, µg/mL) | Antibiotic (MIC, µg/mL) | FIC Index |
|---|---|---|---|---|---|
| | Peptide | Antibiotic | | | |
| ***S. aureus* 949987** | | | | | |
| Macropin-Gentamycin | 8 | 1 | 8 | >64 | 1.02 |
| Macropin -Tobramycin | 8 | 1 | 8 | >64 | 1.02 |
| Macropin -Ciprofloxacin | 2 | 16 | 8 | 16 | 1.25 |
| Macropin -Oxacillin | 4 | 1 | 8 | >64 | 0.52 |
| Macropin -Piperacillin | 8 | 1 | 8 | >64 | 1.02 |
| Macropin -Levofloxacin | 4 | 4 | 8 | 8 | 1 |
| ***S. aureus* 254422** | | | | | |
| Macropin-Gentamycin | 8 | 2 | 8 | >64 | 1.03 |
| Macropin -Tobramycin | 8 | 1 | 8 | >64 | 1.02 |
| Macropin -Ciprofloxacin | 4 | 16 | 8 | 16 | 1.5 |
| Macropin -Oxacillin | 8 | 1 | 8 | >64 | 1.02 |
| Macropin -Piperacillin | 4 | 32 | 8 | >64 | 1 |
| Macropin -Levofloxacin | 8 | 2 | 8 | 4 | 1.5 |
| ***S. aureus* 547582** | | | | | |
| Macropin - Gentamycin | 8 | 1 | 8 | >64 | 1.02 |
| Macropin - Tobramycin | 8 | 4 | 8 | >64 | 1.06 |
| Macropin - Ciprofloxacin | 1 | 8 | 8 | 8 | 1.13 |
| Macropin - Oxacillin | 4 | 1 | 8 | >64 | 0.52 |
| Macropin - Piperacillin | 4 | 2 | 8 | >64 | 0.53 |
| Macropin - Levofloxacin | 8 | 2 | 8 | 4 | 1.13 |

Fractional inhibitory concentration (FIC) indices were interpreted as follows: FICi ≤ 0.5 indicates synergy, 0.5 < FICi ≤ 1.0 indicates additive, 1 < FICi ≤ 4 indicates indifference, and FICi > 4 indicates antagonism.

**Table S2. Effect of Macropin in combination with antibiotics against *P. aeruginosa* strains.**

| Peptide-Antibiotic | Best Concentration of Mixture (MIC, µg/mL) | | Peptide (MIC, µg/mL) | Antibiotic (MIC, µg/mL) | FIC Index |
|---|---|---|---|---|---|
| | Peptide | Antibiotic | | | |
| ***P. aeruginosa* 1034** | | | | | |
| Macropin - Gentamycin | 8 | 1 | 8 | 32 | 1.03 |
| Macropin - Tobramycin | 4 | 32 | 8 | 32 | 1.5 |
| Macropin - Ciprofloxacin | 8 | 4 | 8 | >64 | 1.06 |
| Macropin - Oxacillin | 8 | 1 | 8 | >64 | 1.02 |
| Macropin - Piperacillin | 8 | 1 | 8 | >64 | 1.02 |
| Macropin - Levofloxacin | 8 | 2 | 8 | >64 | 1.03 |
| ***P. aeruginosa* 3320** | | | | | |
| Macropin - Gentamycin | 4 | 1 | 8 | 32 | 0.53 |
| Macropin - Tobramycin | 4 | 1 | 8 | 64 | 0.52 |
| Macropin - Ciprofloxacin | 8 | 1 | 8 | >64 | 1.01 |
| Macropin - Oxacillin | 4 | 1 | 8 | >64 | 0.52 |
| Macropin - Piperacillin | 4 | 1 | 8 | >64 | 0.52 |
| Macropin - Levofloxacin | 8 | 1 | 8 | >64 | 1.01 |
| ***P. aeruginosa* 3241** | | | | | |
| Macropin - Gentamycin | 4 | 1 | 4 | 32 | 1.06 |
| Macropin - Tobramycin | 4 | 1 | 4 | 16 | 1.03 |
| Macropin - Ciprofloxacin | 4 | 1 | 4 | 64 | 1.01 |
| Macropin - Oxacillin | 4 | 4 | 4 | >64 | 1.06 |
| Macropin - Piperacillin | 4 | 1 | 4 | >64 | 1.01 |
| Macropin - Levofloxacin | 4 | 1 | 4 | >64 | 1.01 |

Fractional inhibitory concentration (FIC) indices were interpreted as follows: FICi ≤ 0.5 indicates synergy, 0.5 < FICi ≤ 1.0 indicates additive, 1 < FICi ≤ 4 indicates indifference, and FICi > 4 indicates antagonism.
